# Diabetes mellitus is an independent risk factor for surgical site infection after orthopedic Class I incision surgery: a retrospective cohort study

**DOI:** 10.3389/fsurg.2026.1913043

**Published:** 2026-09-14

**Authors:** Bei Zhong, Dan Zou, Yu Liu

**Affiliations:** 1 Department of Endocrinology, Sir Run Run Hospital, Nanjing Medical University, Nanjing, Jiangsu, China; 2 Department of Geriatrics, Changzhou Geriatric Hospital Affiliated to Soochow University, Changzhou, Jiangsu, China

**Keywords:** Class I incision, diabetes mellitus, firth penalized logistic regression, orthopedic surgery, pathogen distribution, risk factors, surgical site infection

## Abstract

Surgical site infection (SSI) is one of the most devastating complications following orthopedic Class I incision surgery. Diabetes mellitus (DM) has been implicated as an important potential risk factor, but previous studies have yielded conflicting results, largely due to the widespread use of traditional logistic regression for rare event analysis and inadequate control for confounding by surgical procedure type. This retrospective cohort study included 5,845 consecutive patients who underwent orthopedic Class I incision surgery at a tertiary hospital in Nanjing between January 2021 and December 2025. Patients were stratified into DM and non-DM groups. Stratified analysis and Firth penalized maximum likelihood logistic regression were used to control for confounding factors. Four hierarchically adjusted models were constructed to evaluate the stability of the effect of DM, and the pathogenic bacteria spectrum of SSI in our institution was characterized. The overall incidence of SSI was 0.43%. The SSI rate was significantly higher in the DM group than in the non-DM group (1.09% vs. 0.37%, relative risk (*RR*) = 2.93, 95% confidence interval (95% *CI*): 1.11–7.78, *p* = 0.042). After adjusting for demographic characteristics, perioperative factors and surgical procedure category across four hierarchically structured Firth penalized logistic regression models, DM remained a stable independent risk factor for SSI, with odds ratio (OR) values ranging from 3.17 to 3.51 (all *p* < 0.05). In the fully adjusted model, DM was associated with a 3.35-fold increased risk of SSI (*OR* = 3.35, 95% *CI*: 1.11–8.76, *p* = 0.034). Among the 24 isolated pathogenic strains, gram-positive cocci and gram-negative bacilli each accounted for 45.8%. DM is an independent and stable risk factor for SSI after orthopedic Class I incision surgery, and its effect remains significant even after adequate adjustment for surgical procedure type. Gram-positive cocci and gram-negative bacilli are equally prevalent among SSI pathogens in our region, and empirical antimicrobial prophylaxis should provide coverage for both categories of pathogens.

## 1 Introduction

Surgical site infection (SSI) is one of the most common and devastating complications following orthopedic surgery, accounting for approximately 20% of all healthcare-associated infections in surgical patients globally (1–3). Given that orthopedic procedures frequently involve implant placement and require longer operative durations, they carry a significantly elevated risk of SSI (4, 5). A global meta-analysis has demonstrated that the prevalence of incisional SSI following orthopedic surgery ranges from 0.3% to 29% (6), while the prevalence of SSI in diabetic patients undergoing knee arthroplasty can be as high as 5.1% (7). SSI can lead to catastrophic outcomes including implant failure, sepsis, and amputation, resulting in a 2- to 3-fold increase in mortality and imposing a substantial economic burden on both patients and healthcare systems (8–11).

Diabetes mellitus (DM) is widely recognized as an important host factor for postoperative infections (12, 13). Chronic hyperglycemia impairs neutrophil chemotaxis, phagocytosis, and intracellular killing, compromising the host's ability to clear intraoperative bacterial contamination (14, 15). Although multiple cohort studies have identified DM as an independent risk factor for orthopedic SSI (6, 16), conflicting results have been reported in specialized populations such as those undergoing foot and ankle surgery, where several studies failed to demonstrate a significant independent association after multivariable adjustment (17–20).

SSI after orthopedic Class I incision surgery is a rare event (incidence <1%) (21). Orthopedic Class I incision refers to elective clean surgical wounds without gastrointestinal, respiratory or genitourinary tract contamination, representing the largest proportion of orthopedic operations in routine clinical practice (22, 23). Though these wounds are defined as clean and carry baseline low infection risk, most orthopedic Class I procedures require implant placement, which inherently elevates latent SSI risk.

Prior observational studies commonly mixed Class I, II and III orthopedic wounds together without stratified adjustment, creating unmeasured confounding when evaluating the association between DM and postoperative infection­­—this is the key literature gap the present study addresses. Traditional maximum likelihood estimation may produce biased coefficient estimates or fail to converge entirely due to complete or quasi-complete separation (24, 25).

Furthermore, most studies have not adequately controlled for confounding by surgical procedure type, a critical confounder: patients with DM are more likely to undergo higher-risk complex procedures such as spinal surgery and total joint arthroplasty, and this unequal distribution may lead to overestimation of the effect of DM. Notably, even though clean Class I wounds constitute the majority of orthopedic operations and feature a population-wide low baseline SSI rate, diabetes acts as an independent modifying factor that substantially amplifies individual infection risk within this general low-risk surgical subgroup, though this association may lose statistical significance in specific foot and ankle subgroups after multivariable adjustment for comorbidities and surgical factors (19).

To address these methodological gaps, we conducted a retrospective cohort study of 5,845 consecutive patients who underwent orthopedic Class I incision surgery at a tertiary hospital in Nanjing between January 2021 and December 2025, using Firth penalized maximum likelihood logistic regression specifically designed for rare event data (26). The specific aims of this study were: (1) to evaluate the independent association between DM and SSI after orthopedic Class I incision surgery after adjusting for key confounders including age, gender, American Society of Anesthesiologists (ASA) physical status, operative duration, hospital stay, and surgical procedure type; (2) to assess the stability of the effect of DM across four hierarchically adjusted models; and (3) to characterize the pathogenic bacteria spectrum of orthopedic SSI in our institution. Our findings will provide evidence-based guidance for the development of localized perioperative infection prevention strategies.

## 2 Methods

### 2.1 Study design and setting

This retrospective cohort study was conducted at The Affiliated Sir Run Run Hospital of Nanjing Medical University, a 1,200-bed tertiary teaching hospital in Nanjing, Jiangsu Province, China. The study protocol was approved by the Institutional Review Board of Sir Run Run Hospital, Nanjing Medical University, (approval number: 2026-SR-039). Informed consent was waived due to the retrospective nature of the study and the use of fully de-identified electronic medical records.

### 2.2 Study population

We included all consecutive patients who underwent orthopedic Class I incision surgery between January 1, 2021, and December 31, 2025. Class I incision surgery was defined as elective surgery performed on non-infected, non-traumatized tissue without violation of the gastrointestinal, respiratory or genitourinary tracts, and without placement of a drain (23, 27). Patients were excluded if they had a pre-existing infection at the surgical site, underwent emergency surgery, or did not complete the planned surgical procedure. A total of 5,845 patients were included in the final analysis.

### 2.3 Data collection

Data for this retrospective cohort study were retrieved from two integrated institutional electronic sources: (1) real-time nosocomial infection surveillance records, which were prospectively generated as part of routine hospital infection control practice, and (2) electronic medical records containing perioperative clinical variables, which were retrospectively extracted for the purpose of this study.

All electronic medical records and real-time nosocomial surveillance data were automatically de-identified by the hospital information system at the source before data extraction. No names, ID numbers, phone numbers or residential addresses were exported to the research database. The prospective infection surveillance module only recorded aggregated clinical indicators without personal identifiers, which were merged with de-identified operative records for this retrospective cohort analysis.

The prospective nosocomial infection surveillance followed a standardized dual-verification workflow integrated into routine hospital practice. During each patient's hospitalization, attending physicians and ward nurses prospectively documented wound appearance, body temperature, laboratory culture results and all suspected SSI events in the electronic medical record system on a daily basis, which was fully interfaced with the hospital's dedicated nosocomial infection surveillance platform. Attending physicians were required to report or rule out suspected SSI events through the system daily. Subsequently, dedicated infection control physicians from the hospital infection prevention department independently reviewed all reported cases and confirmed or excluded SSI diagnoses according to Centers for Disease Control and Prevention (CDC) criteria on a daily basis (28). This dual-verification surveillance process was standard routine hospital quality management work, conducted independently of and prior to the present retrospective study.

The full study dataset was electronically exported from the hospital-integrated clinical and nosocomial infection surveillance information system described above. Automated consistency checks were applied to the entire dataset to identify missing values, out-of-range entries, and logical contradictions. To validate the accuracy of electronically exported data, a random 10% sample was independently re-reviewed by two trained research assistants against original electronic medical records, focusing on key variables including diabetes mellitus status, SSI diagnosis, operative duration, ASA physical status classification, length of hospital stay, and surgical procedure category. Discrepancies were resolved by consensus or adjudicated by the senior author. Excellent inter-rater agreement was demonstrated, with kappa coefficients of 0.92 for diabetes mellitus diagnosis and 0.95 for SSI diagnosis.

The following variables were collected: (1) demographic characteristics: age at surgery, gender; (2) perioperative variables: operative duration (defined as the time from skin incision to skin closure), ASA physical status classification, length of hospital stay; (3) comorbidities: diabetes mellitus (DM) was identified using International Classification of Diseases, Tenth Revision (ICD-10) codes E10–E14 and confirmed by documentation of antidiabetic medication use or preoperative fasting blood glucose ≥7.0 mmol/L; (4) Surgical information: All procedures were classified into 7 major categories based on electronic medical records: fracture reduction and internal fixation, internal fixation removal, total joint arthroplasty, spinal surgery, arthroscopic minimally invasive surgery, bone and soft tissue repair and debridement surgery, and other orthopedic procedures. This classification was used to control for confounding by surgical procedure type.

These seven categories were defined based on anatomical site, operative complexity, implant requirement and expected baseline SSI risk: fracture reduction and internal fixation and spinal surgery typically involve implant placement and longer operative time; total joint arthroplasty carries a well-documented elevated periprosthetic infection risk (29, 30); internal fixation removal and arthroscopic minimally invasive surgery are generally shorter, lower-risk procedures; bone and soft tissue repair/debridement covers heterogeneous soft-tissue procedures; and the remaining less frequent operations were grouped as “other orthopedic procedures.”

Surgical procedure type was identified as an *a priori* confounder based on established clinical and epidemiological evidence: different orthopedic procedure categories carry substantially different baseline SSI risks, and patients with DM are clinically more likely to require complex, higher-risk procedures such as spinal surgery and total joint arthroplasty due to diabetes-related degenerative spinal disease and arthropathy. This unequal distribution of procedure types between DM and non-DM patients, if left unadjusted, could confound the estimated association between DM and SSI.

### 2.4 SSI definition and ascertainment

SSI was diagnosed according to the Guidelines for the Diagnosis and Management of Surgical Site Infection issued by the Chinese Center for Disease Control and Prevention (2021), which is fully consistent with the US CDC National Healthcare Safety Network (NHSN) definition (31, 32). In accordance with this definition, all three CDC/NHSN SSI subtypes were eligible for inclusion: superficial incisional SSI (involving only skin and subcutaneous tissue), deep incisional SSI (involving deep soft tissues such as fascia and muscle layers), and organ/space SSI (involving any anatomical structure other than the incision that was opened or manipulated during surgery). No SSI subtype was excluded from the analysis.

Consistent with the routine hospital-wide nosocomial surveillance workflow described in Section 2.3, all suspected SSI events were initially identified during the index hospitalization, standardized postoperative outpatient follow-up and inpatient re-admission encounters, and were first reported by attending surgical clinicians. All suspected cases were subsequently and independently reviewed and confirmed by two board-certified infection-control physicians from the hospital infection prevention department. These physicians evaluated cases based on wound manifestations, fever records, laboratory results, and microbial culture data, without routine notification of patients' preoperative diabetes status during diagnostic adjudication. No research team members participated in any SSI diagnostic decisions.

Postoperative surveillance relied entirely on standardized routine in-person outpatient follow-up. Patients with abnormal wound conditions or suspected postoperative infection were routinely readmitted for further evaluation and treatment. The hospital nosocomial infection surveillance system automatically captures all suspected infection events within one year after orthopedic implant surgery and links each infection record to the index operative procedure, enabling systematic, passive, full-cycle postoperative surveillance without additional manual research-driven follow-up. In accordance with official guidelines, all patients were monitored for SSI within the standard 30-day postoperative window, while surveillance was extended to one year for procedures involving permanent implant placement. All outcome data were retrospectively retrieved from the integrated institutional electronic medical and nosocomial surveillance system for the entire 5,845-patient cohort, with follow-up observation truncated uniformly on the data extraction date. Patients without a full 1-year observation window were classified based on all available surveillance records up to the cutoff date, and no patients were excluded due to incomplete follow-up.

### 2.5 Statistical analysis

Statistical analysis was performed using R software version 4.4.2 (R Foundation for Statistical Computing, Vienna, Austria). Categorical variables are presented as frequencies (percentages) and compared using the chi-square test or Fisher's exact test, as appropriate. Continuous variables were tested for normality using the Shapiro–Wilk test. Non-normally distributed continuous variables are presented as medians (interquartile ranges, IQR) and compared using the Mann–Whitney U test.

SSI incidence rates were calculated for the DM and non-DM groups stratified by surgical procedure type, and between-group comparisons were performed using Fisher's exact test. Given the rare nature of SSI and the presence of zero-event cells in several surgical procedure subgroups, the generalized Cochran-Mantel-Haenszel test with Monte Carlo approximation was used to assess the association between DM and SSI after stratifying by surgical procedure category.

Given the well-documented rare nature of SSI in clean orthopedic surgery (21), traditional maximum likelihood logistic regression may produce biased coefficient estimates or fail to converge due to complete or quasi-complete separation. Therefore, Firth penalized maximum likelihood logistic regression was used for multivariable analysis. This method reduces bias by adding a penalty term proportional to Jeffreys invariant prior to the likelihood function (33, 34), and is the recommended approach for risk factor analysis of rare events.

Variable inclusion for multivariable modelling was guided by *a priori* clinical and epidemiological knowledge of SSI risk factors derived from existing literature, supplemented by baseline between-group comparisons. Variables that were either clinically well-established risk factors for orthopedic SSI or showed statistically significant baseline imbalance between DM and non-DM groups (*p* < 0.05) were considered for inclusion in the multivariable models. Formal directed acyclic graph (DAG) analysis was not performed in this study. Automated stepwise selection or purely *p*-value-driven variable screening was avoided throughout model development (35, 36). Four hierarchically adjusted models were constructed to disentangle and verify the stability of the independent effect of DM on SSI:

Model 1 (crude model): DM only
Model 2: adjusted for demographic characteristics (age and gender)
Model 3: additionally adjusted for patient baseline health status and surgical factors (ASA ≥3, operative duration ≥2 h, and hospital stay)
Model 4 (fully adjusted model): additionally adjusted for surgical procedure type on the basis of Model 3

Odds ratios (OR) and 95% confidence intervals (CI) were calculated for each variable. Multicollinearity between independent variables was tested using variance inflation factor (VIF) analysis. A VIF value ≥5 was considered indicative of significant multicollinearity. All statistical tests were two-sided, and a *p*-value <0.05 was considered statistically significant.

## 3 Results

### 3.1 Baseline characteristics

A total of 5,845 patients were included in the final analysis, of whom 459 (7.9%) had diabetes mellitus (DM) and 5,386 (92.1%) did not. The baseline characteristics of the two groups are presented in Table 1. Patients in the DM group were significantly older than those in the non-DM group (median 62 years vs. 52 years, *p* < 0.001). The DM group also had a higher proportion of patients with ASA physical status ≥3 (19.8% vs. 4.9%, *p* < 0.001), longer median operative duration (1.3 h vs. 1.2 h, *p* = 0.018), and longer median hospital stay (10 days vs. 8 days, *p* < 0.001). A significant difference in gender distribution was observed between the two groups (*p* < 0.001).

**Table 1:** Baseline characteristics of patients with and without diabetes mellitus.

Characteristic	Total (*N* = 5,845)	DM (*N* = 459)	Non-DM (*N* = 5,386)	*p* value
Age (years), median (IQR)	53 (35–63)	62 (56–72)	52 (34–61)	<0.001
Gender, *n*(%)				<0.001
Male	3,019 (51.7)	202 (44.0)	2,817 (52.3)	
Female	2,826 (48.3)	257 (56.0)	2,569 (47.7)	
ASA≥3, *n*(%)	357 (6.1)	91 (19.8)	266 (4.9)	<0.001
Operative duration ≥2 h, *n*(%)	1,270 (21.7)	118 (25.7)	1,152 (21.4)	0.036
Operative duration (h), median (IQR)	1.2 (0.8–1.8)	1.3 (0.8–2)	1.2 (0.8–1.8)	0.018
Hospital stay (days), median (IQR)	8 (5–12)	10 (7–15)	8 (5–12)	<0.001
Surgical procedure category, n(%)				<0.001
Fracture reduction and internal fixation	2,229 (38.14)	147 (32.03)	2,082 (38.66)	
Internal fixation removal	1,456 (24.91)	75 (16.34)	1,381 (25.64)	
Arthroscopic minimally invasive surgery	565 (9.67)	38 (8.28)	527 (9.78)	
Spinal surgery	441 (7.54)	65 (14.16)	376 (6.98)	
Bone and soft tissue debridement/repair	330 (5.65)	27 (5.88)	303 (5.63)	
Total joint arthroplasty	318 (5.44)	57 (12.42)	261 (4.85)	
Other orthopedic procedures	506 (8.66)	50 (10.89)	456 (8.47)	

The distribution of surgical procedure categories differed significantly between the DM and non-DM groups (Fisher's exact test, *p* < 0.001). Patients with DM were more likely to undergo spinal surgery (14.16% vs. 6.98%) and total joint arthroplasty (12.42% vs. 4.85%), but less likely to undergo internal fixation removal (16.34% vs. 25.64%), indicating that surgical procedure type was an important potential confounder.

### 3.2 Incidence of surgical site infection

Overall, 25 cases of surgical site infection (SSI) were identified, corresponding to an overall incidence of 0.43%. The SSI rate was significantly higher in the DM group (1.09%, 5/459) than in the non-DM group (0.37%, 20/5386), with a relative risk (RR) of 2.93 (95% CI 1.11–7.78, *p* = 0.042) (Table 2).

**Table 2:** Comparison of SSI rates between patients with and without diabetes mellitus.

Group	Infected	Non-infected	Total	Infection rate (%)	RR (95% *CI*)	*p* value
DM	5	454	459	1.09	2.93 (1.11–7.78)	0.042
Non-DM	20	5,366	5,386	0.37	1 (Ref)	—

### 3.3 Stratified analysis of SSI by surgical procedure category

Stratified analysis by surgical procedure category showed that SSI rates were consistently higher in DM patients across several subgroups, including internal fixation removal, spinal surgery, and fracture reduction and internal fixation. However, no statistically significant differences were observed within any individual subgroup (all *p* > 0.05). Zero-event cells were present in several subgroups, consistent with the rare event nature of SSI in Class I orthopedic surgery (Table 3).

**Table 3:** SSI rates by surgical procedure category and diabetes status.

Surgical procedure category	DM group		Non-DM group		*p* value
	Total	SSI *n*(%)	Total	SSI *n*(%)	
Total joint arthroplasty	57	0 (0)	261	0 (0)	1
Arthroscopic surgery	38	0 (0)	527	1 (0.19)	1
Internal fixation removal	75	1 (1.33)	1,381	4 (0.29)	0.233
Spinal surgery	65	2 (3.08)	376	2 (0.53)	0.105
Fracture reduction and internal fixation	147	2 (1.36)	2,082	10 (0.48)	0.185
Bone and soft tissue debridement/repair	27	0 (0)	303	3 (0.99)	1
Other orthopedic procedures	50	0 (0)	456	0(0)	1

### 3.4 Stratified analysis by surgical procedure type

To evaluate whether the association between DM and SSI persisted after accounting for differences in surgical procedure type, a stratified analysis was conducted across the seven procedure categories. Given the presence of zero-event cells in several subgroups, the generalized Cochran-Mantel-Haenszel test with Monte Carlo approximation was applied. After adjustment for surgical procedure type, DM remained significantly associated with SSI (*χ^2^* = 5.27, *df* = 1, *p* = 0.041), with a pooled odds ratio of 3.10 (95% *CI*: 1.13–8.49).

### 3.5 Multivariable firth penalized logistic regression

Four hierarchically adjusted models were constructed to evaluate the stability of the effect of DM:

Model 1 (crude model): DM only
Model 2: adjusted for age and sex
Model 3: additionally adjusted for ASA ≥3, operative duration ≥2 h, and hospital stay
Model 4 (fully adjusted model): additionally adjusted for surgical procedure category

Univariate Firth regression identified DM (*OR* = 3.17, 95% *CI* 1.11–7.63, *p* = 0.033), operative duration ≥2 h (*OR* = 2.45, 95% *CI* 1.12–5.38, *p* = 0.032), and longer hospital stay (*OR* = 1.03, 95% *CI* 1.02–1.04, *p* < 0.001) as significant predictors of SSI.

The results of multivariable analysis are presented in Table 4. DM remained an independent risk factor for SSI across all four models, with OR values ranging from 3.17 to 3.51 (all *p* < 0.05), indicating that the effect of DM was highly stable and independent of other confounding factors. In the fully adjusted model 4, DM was associated with a 3.35-fold increased risk of SSI (*OR* = 3.35, 95% *CI* 1.11–8.76, *p* = 0.034).

**Table 4:** Effect estimates for DM across four hierarchically adjusted firth regression models.

Model	Variables included	*OR*	95% *CI*	*p* value
Model 1	DM only	3.168	1.107–7.631	0.033
Model 2	DM + age + sex	3.271	1.095–8.451	0.035
Model 3	DM + age + sex + ASA ≥3 + operative duration ≥2 h + hospital stay	3.505	1.169–9.098	0.027
Model 4	Model 3 + surgical procedure category	3.353	1.110–8.759	0.034

Longer hospital stay was independently associated with increased SSI risk, with each additional day of hospitalization associated with a 3.2% increase in risk (*OR* = 1.032, 95% *CI* 1.016–1.047, *p* = 0.001). Age, sex, ASA ≥3, operative duration ≥2 h, and most surgical procedure categories were not independently associated with SSI. Only other orthopedic procedures had a significantly lower risk of SSI compared to the reference group (bone and soft tissue debridement/repair) (*OR* = 0.093, 95% *CI* 0.001–0.989, *p* = 0.049).

### 3.6 Pathogen distribution

A total of 24 pathogenic isolates were cultured from 25 SSI episodes, with one case yielding negative cultures. Gram-positive cocci and gram-negative bacilli each accounted for nearly half of all isolates (45.8% each). The most frequently recovered organisms included *Enterobacter spp.*, *Staphylococcus aureus*, *Klebsiella pneumoniae*, and *Enterococcus spp*.

## 4 Discussion

### 4.1 Main findings

The overall incidence of SSI in this cohort of orthopedic Class I incision surgery was 0.43%, consistent with low SSI rates observed in earlier studies focusing on clean orthopedic operations (21). First, all included surgeries were Class I clean operations without preoperative infection or gastrointestinal/respiratory/genitourinary tract contamination, which inherently carry a low baseline infection risk. Second, our hospital implements standardized perioperative infection control protocols, including appropriate prophylactic antibiotic administration, strict aseptic technique and dedicated operating-room infection management. Third, comprehensive SSI surveillance was conducted throughout the study period: in-hospital daily prospective monitoring was performed by attending clinicians and infection-control physicians, and post-discharge surveillance relied on routine outpatient follow-up combined with the hospital's automated nosocomial infection surveillance system, which captures all infection events linked to the index operation within one year for implant-related procedures. Importantly, our surveillance included all CDC-defined SSI categories—superficial incisional, deep incisional and organ/space infections—without excluding mild superficial cases. Therefore, the low SSI incidence observed in this study reflects the true low baseline infection rate of clean orthopedic surgery under standardized infection control, rather than underreporting or selective exclusion of superficial infections.

In this retrospective cohort study of 5,845 consecutive patients who underwent orthopedic Class I incision surgery at a tertiary hospital in Nanjing over a 5-year period, our results demonstrated that DM is an independent risk factor for SSI after orthopedic Class I incision surgery, with a 3.35-fold increased risk after adjustment for all known confounders. Notably, the effect of DM was highly stable across four hierarchically adjusted models. Additionally, longer hospital stay was independently associated with SSI risk, and the pathogen spectrum of orthopedic SSI in our institution showed an equal distribution of gram-positive cocci and gram-negative bacilli.

The overall prevalence of diabetes mellitus in our orthopedic Class I incision cohort was 7.9%, which is consistent with the local middle-aged and elderly population metabolic disease prevalence in eastern China (37). This relatively low proportion is attributable to our sample being restricted to elective clean Class I orthopedic surgery; patients with severe multi-system comorbidities or advanced diabetic complications were more likely to receive delayed or staged surgery and therefore not captured within this consecutive surgical sample.

### 4.2 Association between diabetes mellitus and SSI

Our finding that DM is an independent risk factor for orthopedic SSI aligns with most previous cohort studies (16), while some studies focusing on foot and ankle surgery have reported non-significant associations after covariate adjustment (19). A key methodological strength of the present work is the comprehensive adjustment for surgical procedure type, an important potential confounder, via both stratified analysis and multivariable regression models. Prior research has observed that patients with DM tend to receive higher-risk complex operations including spinal surgery and total joint arthroplasty, and unaccounted imbalance in surgical categories may overestimate the magnitude of DM's association with SSI. In our analysis, DM retained a statistically significant relationship with SSI after accounting for surgical procedure type, with OR estimates ranging from 3.17 to 3.51, which supports an independent effect of diabetes itself on postoperative infection risk. Notably, the effect estimate from this stratified analysis (pooled OR = 3.10) was highly consistent with that from the fully adjusted Firth regression model (OR = 3.35), providing cross-validation of the robustness of the association between DM and SSI.

The biological mechanisms linking diabetes to impaired surgical wound healing are dual-driven by chronic insulin-resistant disease status and acute perioperative hyperglycemia (38, 39). First, inherent insulin resistance in DM disrupts inflammatory signaling, neutrophil chemotaxis, phagocytosis, and intracellular bacterial clearance capacity, independent of transient elevated blood glucose levels (15, 40, 41). Accumulated advanced glycation end products (AGEs) further trigger endothelial dysfunction, reducing tissue perfusion and oxygen supply at the surgical incision site. Second, poor glycemic control amplifies these immune defects: elevated preoperative fasting glucose and elevated HbA1c exacerbate local tissue inflammation and bacterial proliferation, substantially elevating SSI risk (42, 43). Notably, not all patients with DM present uncontrolled glycemia, and well-managed diabetic patients carry markedly lower infection risk compared to those with sustained hyperglycemia (44). Although we could not stratify analyses by continuous HbA1c or graded glycemic status due to incomplete standardized glycemic records in our retrospective dataset, the independent association of DM itself suggests that the underlying metabolic disease may confer intrinsic infection vulnerability that is not fully explained by acute perioperative hyperglycemia alone. Furthermore, DM is frequently accompanied by obesity and peripheral vascular disease, which further impair wound healing and innate host defense against pathogens.

### 4.3 Analysis of other risk factors

Operative duration ≥2 h was significantly associated with SSI in univariate analysis but lost statistical significance after multivariable adjustment, likely because its effect was attenuated after adjusting for hospital stay, rather than due to insufficient statistical power, given the relatively low proportion of procedures exceeding 2 h in our cohort (median operative duration 1.2 h).

Longer hospital stay was the only factor other than DM independently associated with SSI risk, with each additional day of hospitalization associated with a 3.2% increase in risk. However, the retrospective design precludes causal inference: longer hospital stay may increase nosocomial cross-infection risk, but incipient SSI may also prolong hospitalization for treatment. Prospective studies are needed to clarify this relationship.

ASA physical status ≥3 was not significantly associated with SSI, consistent with some prior reports (45) but differing from others (46). This may reflect the low prevalence of ASA ≥3 patients (6.1%) in our cohort, more intensive perioperative optimization for higher-comorbidity patients (47, 48), or potential mediating pathways, whereby ASA status may influence SSI risk via operative duration or hospital stay.

### 4.4 Pathogen distribution

Our results showed that gram-positive cocci and gram-negative bacilli each accounted for 45.8% of SSI pathogens in our institution, which differs from the traditional perception that gram-positive bacteria predominate in orthopedic SSI (11, 49). This difference may reflect regional variations in pathogen epidemiology and antibiotic prescribing practices. The most common pathogens isolated were Enterobacter spp., Staphylococcus aureus, Klebsiella pneumoniae, and Enterococcus spp.

This finding has important implications for empirical antimicrobial prophylaxis. Traditionally, first-generation cephalosporins, which primarily cover gram-positive bacteria, are recommended for orthopedic Class I incision surgery (50). However, our results suggest that in institutions with a high prevalence of gram-negative SSI, empirical prophylaxis should also provide coverage against common gram-negative pathogens to improve preventive efficacy.

### 4.5 Novel contributions and methodological strengths

Three key novel observations from this work supplement existing orthopedic SSI literature. First, we provide evidence that DM acts as an independent SSI risk factor restricted to homogeneous orthopedic Class I clean surgical cohorts; many prior investigations combined mixed wound classifications (Class I, II and III), which complicates specific evaluation of DM's effect among low-risk clean-incision patients. Second, utilizing Firth penalized maximum likelihood logistic regression across four hierarchically adjusted models, we observe consistent DM-associated OR estimates between 3.17 and 3.51 after full adjustment for surgical category. This analytical approach mitigates sparse-data bias common to conventional logistic regression used in earlier SSI studies. Third, we report the local pathogen distribution of orthopedic SSI at a tertiary eastern China hospital, with gram-positive cocci and gram-negative bacilli each accounting for 45.8% of isolates. This local distribution differs from the widely cited predominance of gram-positive organisms in Western cohorts and provides reference for institutional empirical antibiotic selection.

This study has several important methodological strengths. First, it included a large sample of consecutive patients with high-quality data, extracted independently by two trained research assistants with excellent inter-rater reliability. Second, we used Firth penalized logistic regression instead of traditional logistic regression to address the rare nature of SSI, effectively avoiding small sample bias and model non-convergence. Third, we constructed four hierarchically adjusted models to clearly demonstrate the stability of the effect of DM, greatly enhancing the persuasiveness of our conclusions. Fourth, we controlled for confounding by surgical procedure type using both stratified analysis (generalized Cochran-Mantel-Haenszel test) and multivariable regression, addressing the core methodological limitation of previous similar studies.

### 4.6 Limitations

This study has several notable limitations that should be acknowledged for proper interpretation of results.

First, it was a single-center retrospective study, and findings may not be fully generalizable to other medical centers with distinct perioperative infection control protocols and local pathogen epidemiology.

Second, SSI ascertainment entirely relied on institutional electronic medical and nosocomial surveillance records; infectious complications exclusively managed at external hospitals could not be captured by our internal monitoring system. For patients receiving implant-related orthopedic surgery with an operation date close to the data extraction cutoff, a complete 12-month surveillance window was unavailable, and only infection event records up to the cutoff date were incorporated into analyses.

Third, we lacked standardized continuous HbA1c and serial perioperative blood glucose records to conduct stratified analyses comparing SSI risk across graded glycemic control subgroups, limiting our ability to disentangle the separate effect of underlying diabetic disease and acute hyperglycemia.

Fourth, surgical procedures were categorized into seven distinct subgroups for confounding adjustment, while the total number of observed SSI events remained small (*n* = 25). This imbalance between abundant categorical strata and limited outcome counts may constrain statistical analytical performance, even when applying Firth penalized maximum likelihood logistic regression designed for rare event data.

Fifth, several established SSI risk factors were absent from our dataset, including body mass index, smoking history, detailed prophylactic antibiotic timing and dosage, and specific implant subtypes, which may leave residual unmeasured confounding (24, 51).

Finally, pathogen antibiotic susceptibility testing was not performed for isolated strains, restricting deeper interpretation of targeted antimicrobial treatment strategies. Additionally, SSI subtype distribution (superficial, deep, and organ/space) was not separately recorded in the institutional surveillance database, preventing subtype-specific analysis.

### 4.7 Conclusions

In conclusion, DM is an independent and stable risk factor for SSI after orthopedic Class I incision surgery, with a 3.35-fold increased risk even after adjustment for all known confounders including surgical procedure type. The pathogen spectrum of orthopedic SSI in our institution shows an equal distribution of gram-positive cocci and gram-negative bacilli. These findings suggest that diabetic patients should be prioritized for enhanced perioperative infection prevention measures regardless of the type of orthopedic surgery they undergo. Furthermore, empirical antimicrobial prophylaxis should be tailored to the local pathogen spectrum, with consideration given to covering both gram-positive and gram-negative pathogens.

## Data Availability

The raw data supporting the conclusions of this article will be made available by the authors, without undue reservation.
